# Herbal medicine IMOD suppresses LPS-induced production of proinflammatory cytokines in human dendritic cells

**DOI:** 10.3389/fphar.2015.00064

**Published:** 2015-03-27

**Authors:** Saeedeh Mirzaee, Agata Drewniak, Ramin Sarrami-Forooshani, Tanja M. Kaptein, Farhad Gharibdoost, Teunis B. H. Geijtenbeek

**Affiliations:** ^1^Department of Experimental Immunology, Academic Medical Center, University of AmsterdamAmsterdam, Netherlands; ^2^Rheumatology Research Center, Tehran University of Medical ScienceTehran, Iran

**Keywords:** IMOD, immune-modulation, LPS, pro-inflammatory cytokines, dendritic cells

## Abstract

Traditional medicines that stimulate or modulate the immune system can be used as innovative approaches to treat immunological diseases. The herbal medicine IMOD has been shown to strongly modulate immune responses in several animal studies as well as in clinical trials. However, little is known about the mechanisms of IMOD to modulate immunity. Here we have investigated whether IMOD modulates the immunological function of human dendritic cells (DCs). IMOD alone did not induce DC maturation nor production of cytokines. Notably, IMOD decreased the production of pro-inflammatory cytokines IL-6, IL-12 p70, and TNFα by LPS-activated DCs at both mRNA and protein levels in a dose dependent manner. In contrast, treatment with IMOD did not affect LPS induced-production of the anti-inflammatory cytokine IL-10. Furthermore, IMOD inhibited T cell activation/proliferation by LPS-treated DCs and skewed T-cells responses toward the T helper type 2 polarization. These data strongly indicate that IMOD has a potent immunomodulatory ability that affects TLR signaling and thereby modulates DC function. Insight into the immunomodulatory effect of herbal medicine IMOD may provide innovative strategies to affect the immune system and to help combat various diseases.

## Introduction

Elucidation of the mechanisms of action of herbal medicines is important to understand their therapeutic effects on different diseases and to design successful therapies (Huang et al., 2008). Recently, a new herbal based medicine with immunomodulatory capacities – Setarud (IMOD) – was introduced as an additional therapy in various inflammatory diseases and HIV infection (Mohraz et al., 2009; Mahmoodpoor et al., 2010). IMOD has been shown to affect immune responses in animal studies (Mohammadirad et al., 2011). IMOD consists of a mixture of herbal extracts (*Tanacetum vulgare, Rosa canina,* and *Urtica dioica*) supplemented with selenium. The different herbal ingredients of IMOD possess anti-inflammatory, anti-viral and immune modulating properties; the lectin and polysaccharide fractions of *U. dioica* (nettle) exhibits anti-viral and anti-inflammatory properties (Balzarini et al., 1992; Chrubasik et al., 2007a,b), whereas the extract from *T.* *vulgare* possesses anti-inflammatory properties (Schinella et al., 1998). Beta-caroten of *R. canina* fruits delays the increase of serum blood glucose and cholesterol (Ninomiya et al., 2007). Selenium is an essential trace element that plays a key role in protecting cells from oxidative stress, and selenium supplementation in the diet may reduce the risk of cardiomyopathy, cancer, and immune disorders in humans (Rayman, 2000; Ren et al., 2012).

IMOD treatment positively influences the treatment of patients with severe sepsis by decreasing the levels of tumor necrosis factor (TNF) in their serum (Mahmoodpoor et al., 2010). Additionally, IMOD has also been found to be effective in other types of immune inflammatory-based diseases such as ulcerative colitis and type I diabetes (Mohammadirad et al., 2011). Furthermore, IMOD has a positive influence on the CD4 level in HIV infected individuals (Mohraz et al., 2009, 2013). These preclinical and clinical studies suggest that IMOD limits inflammatory responses but the mechanisms of immunodulation by IMOD remain unknown.

Dendritic cells (DCs) are essential to initiate adaptive immune responses to different pathogens such as bacteria, viruses, and fungi (Banchereau and Steinman, 1998). DCs sample the environment for invading pathogens and interactions with pathogens or pathogenic components leads to DC maturation and consequently migration to the lymphoid tissues, where mature DCs present processed antigens on MHC class II and I molecules to CD4^+^ and CD8^+^ T cells, respectively, thereby activating pathogen-specific T cells. Furthermore, DCs are crucial in instructing CD4^+^ T helper (T_H_) cell polarization, which is paramount to an efficient immune response (Kapsenberg, 2003). T_H_ polarization is driven by secretion of specific cytokines and cell-surface expression of co-stimulatory molecules by DCs in response to infectious pathogens. The pro-inflammatory cytokine IL-12 promotes the development of T_H_1, which secrete IFN-γ and are paramount in the protection against intracellular microorganisms. In contrast a suppression of IL-12 and induction of cytokines such as IL-10 and IL-4 promotes T_H_2 development (Pulendran et al., 2010). T_H_2 cells secrete mainly IL-4 to induce humoral immunity against extracellular pathogens (Chen et al., 2012).

Here, we have investigated the effect of IMOD on DC function and subsequent T_H_ cell polarization. IMOD alone did not affect DC function but our data strongly indicate that IMOD suppressed TLR4-induced pro-inflammatory cytokine production by DCs. Interestingly, IMOD did not affect expression of anti-inflammatory cytokine IL-10. Concomitantly, we observed that IMOD suppressed T cell activation and skewed T_H_ cell polarization toward T_H_2. These data strongly suggest that IMOD decreases DC activation via TLRs, which might explain its immunomodulatory effects in inflammatory diseases. Further research into the exact molecular mechanism of TLR suppression might provide novel targets for immunomodulatory therapies.

## Materials and Methods

### Cell Culture and Dendritic Cell Stimulation

Immature DCs were generated from monocytes obtained from buffy coats of healthy blood donors (Sanquin Bloedbank, Amsterdam, The Netherlands) after culture in presence of IL-4 and GM-CSF for 6 days as described before (Geijtenbeek et al., 2003). The study was approved by the local Medical Ethics Review Committee in accordance with the ethical guidelines of the Academic Medical Center, and Declaration of Helsinki. 100,000 DCs were stimulated with different concentrations of IMOD (1:400, 1:800, 1:1600, 1:3200; stock concentration 30 mg/mL, Pars Roos Co., Teheran, Iran) in the presence or absence of *Escherichia coli* LPS (10 ng/ml, Sigma-Aldrich). For isolation of mRNA, cells were lysed after 6 h of incubation. To measure cytokine production and expression of cell surface markers, cells were incubated for 18 h. IL-6, IL-12p40, IL-10, and TNF-α were measured in culture supernatants by ELISA (Invitrogen) according to the manufacturer’s recommendations DCs were analyzed by flow cytometry analysis (FACS) for expression of CD80, and CD86 (CD80-PE, CD86-PE, Pharmingen).

### RNA Extraction, and Quantitative Real-Time PCR

mRNA was specifically isolated with the mRNA capture kit (Roche) and cDNA was synthesized with the reverse transcriptase kit (Promega). For real-time PCR analysis, PCR amplification was performed in the presence of SYBR green, as previously described (García-Vallejo et al., 2004). Specific primers were designed using Primer Express 2.0 (Applied Biosystems, Table S1; Gringhuis et al., 2007). The Ct value is defined as the number of PCR cycles where fluorescence signal exceeds the detection threshold value. For each sample, the normalized amount of target mRNA Nt was calculated from the obtained Ct values for both target and GAPDH mRNA with Nt = 2^Ct(GAPDH)^^-^^Ct(target)^. The relative mRNA expression was obtained by setting Nt in LPS-stimulated DCs at 1 within one experiment and for each donor.

### T Lymphocyte Proliferation (Mixed Lymphocyte Reaction)

Dendritic cells were pre-incubated with IMOD (concentration 1:800) for 18 h in presence and absence of LPS. Thereafter the DCs were washed to remove remaining IMOD before adding of allogeneic T cells. DCs were cultured with allogeneic peripheral blood lymphocytes (PBL; 1 × 10^5^) at different ratios (1: 50 to 1: 400) for 5 days. T cells proliferation was assessed by measuring the overnight incorporation of BrdU (BrdU labeling reagent, Invitrogen. PE mouse anti BrdU Ab, BD Pharmingen).

### T Cell Differentiation Assay

Highly purified CD4^+^CD45RA^+^CD45RO^-^ naive Th cells (>98% as assessed by flow cytometry) were purified from PBMCs using a human CD4^+^/CD45RO^-^ column kit (R&D Systems, Minneapolis, MN, USA). Immature DCs were stimulated with different concentrations of IMOD (1: 800 and 1: 1600) for 48 h. DCs were washed extensively, and naive CD4^+^ T cells were added to stimulated DCs. On days 6 and 9 of co-culture, cells were stimulated with IL-2 (10 U/ml). On day 13, T cells were restimulated with the phorbol ester PMA (100 ng/ml) and ionomycin (1 μg/ml) in the presence of brefeldin A (10 μg/ml), stained for intracellular IL-4 and IFN-γ with phycoerythrin- and fluorescein isothiocyanate–labeled antibodies (Becton Dickinson), respectively, and analyzed on a FACSCanto (Becton Dickinson).

### Statistics

Statistical analysis was performed using the Student’s *t*-test for paired observations, unless stated otherwise. Comparisons with probability values of less than 0.05 were considered to be significant.

## Results

### IMOD has no Significant Influence on DC Maturation

**FIGURE 1:**
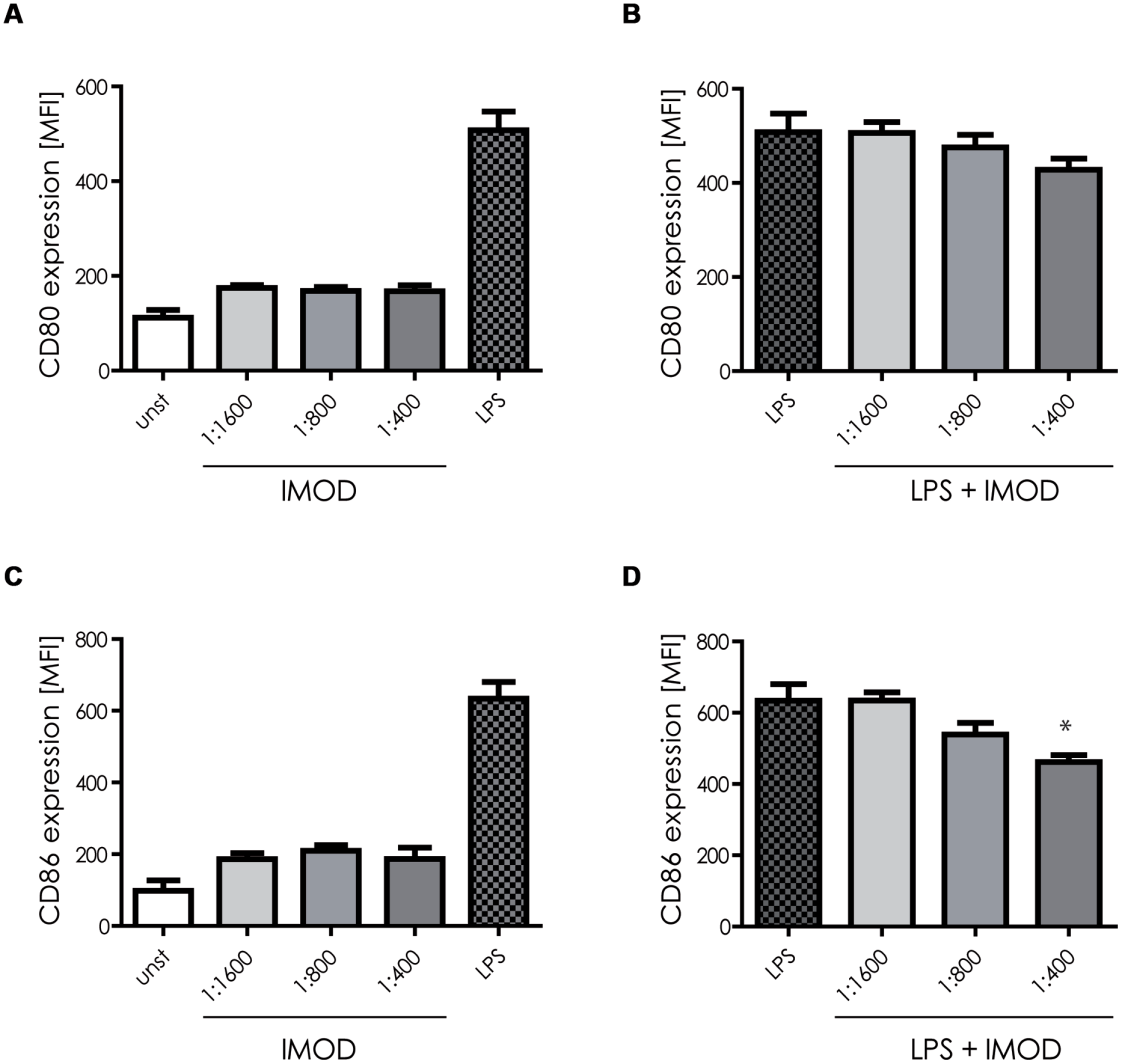
**IMOD treatment does not affect dendritic cell (DC) maturation.** Immature DCs were stimulated with different dilutions of IMOD in the presence or absence of LPS. 18 h after stimulation, expression of maturation markers CD80 **(A,B)** and CD86 **(C,D)** was measured by flow cytometry. The data are presented as mean ± standard deviation from at least three independent experiments. ^∗^*P* < 0.05. IMOD/LPS-treated vs. LPS stimulated samples.

First we investigated the effect of IMOD on DC function. Human monocyte-derived DCs were incubated with different concentrations of IMOD for 18 h and DC maturation was determined by measuring expression of co-stimulatory molecules CD80 and CD86. Concentrations were chosen that did not affect cell viability (Figure S1). IMOD alone did not induce DC maturation, as expression of both maturation markers, CD80 and CD86, on IMOD-treated DCs was similar to that of immature DCs (**Figures 1A,C** and Figure S2). Next, we investigated whether IMOD affects DC maturation induced by TLR4 ligand LPS. IMOD treatment did not affect the LPS-induced expression of maturation markers CD80 and CD86. CD86 expression was decreased only at the highest IMOD concentration (**Figures 1B,D** and Figure S2). Additionally the levels of other maturation markers such as CD83, CD40, or HLA-DR were not affected by IMOD treatment, in either unstimulated or LPS treated cells (Figure S2). These data suggest that IMOD does not affect DC maturation.

### IMOD Suppresses Pro-Inflammatory Cytokine Expression

**FIGURE 2:**
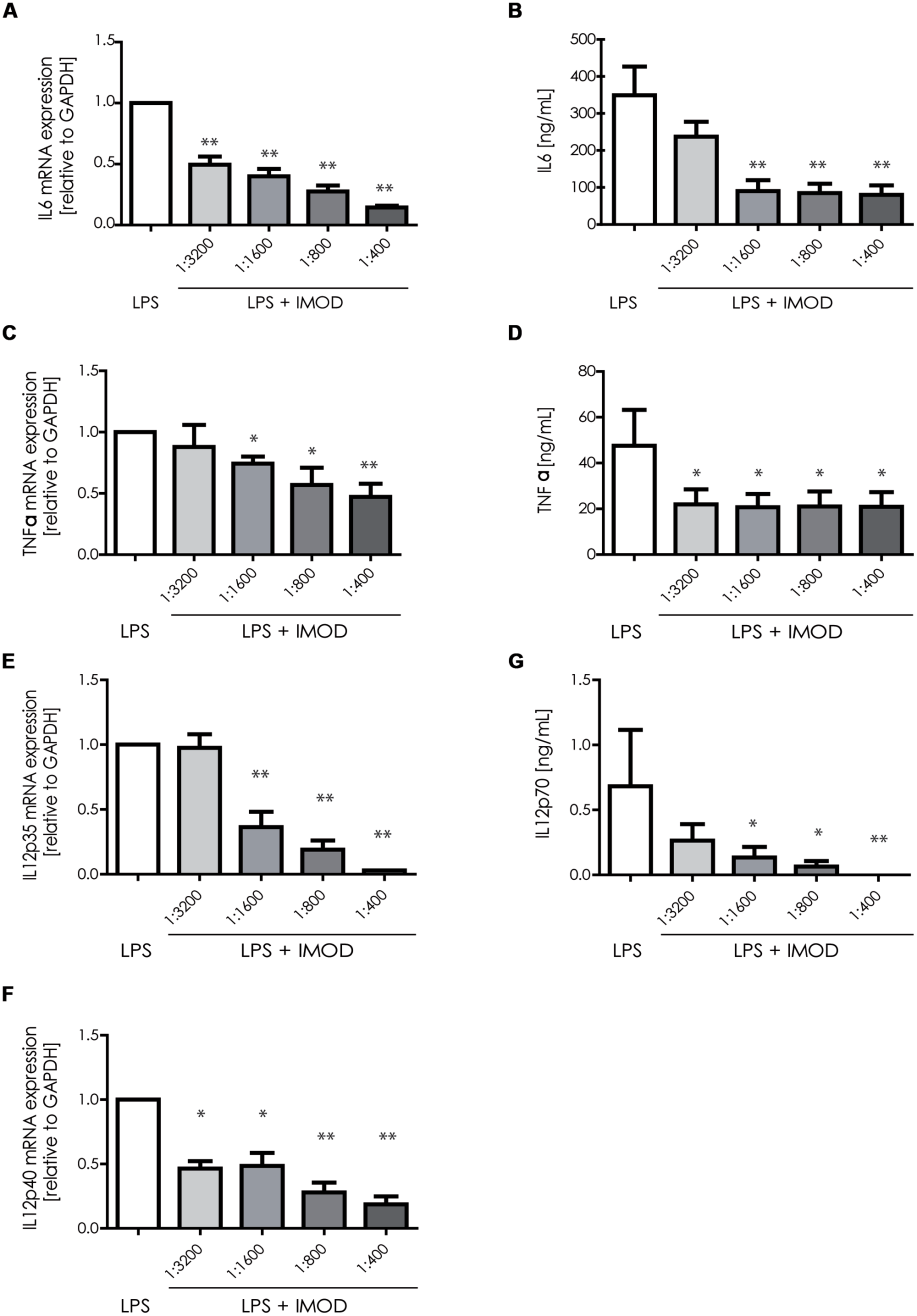
**1IMOD suppresses TLR4-induced pro-inflammatory cytokines.** Immature DCs were stimulated with LPS alone or in combination with IMOD and cytokine expression was measured after 6 and 18 h at mRNA and protein levels, respectively. IMOD suppressed mRNA levels of LPS-induced IL-6 **(A)**, TNFα **(C)**, IL-12p35 **(E),** and IL12p40 **(G)** as measured by quantitative real time PCR. Similarly, IMOD inhibited cytokine production of IL-6 **(B)**, TNFα **(D),** and IL-12p70 **(F)** at protein level as measured by ELISA. The data are represented as mean ± standard deviation of at least three independent experiments. ^∗^*P* < 0.05; ^∗∗^*P* < 0.01. IMOD/LPS-treated vs. LPS stimulated samples.

**FIGURE 3:**
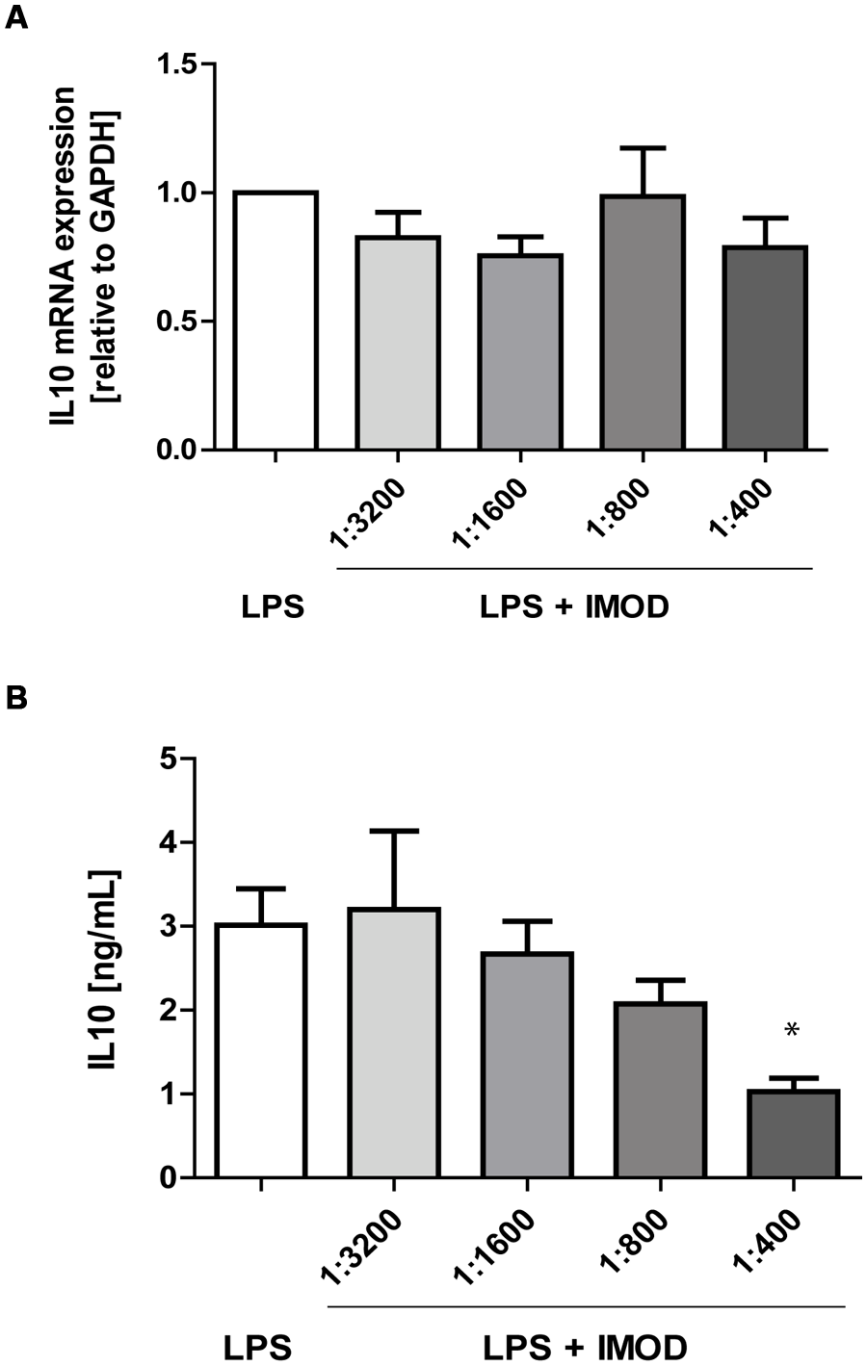
**IMOD does not affect IL-10 expression by LPS-stimulated dendritic cells.** DCs were stimulated with LPS alone or in combination with IMOD and IL-10 expression was measured after 6 and 18 h at mRNA **(A)** and protein level **(B)**, respectively. The data are represented as mean ± standard deviation of at least three independent experiments. ^∗^*P* < 0.05 IMOD/LPS treated vs. LPS stimulated samples.

Next, we investigated whether IMOD induces cytokine production or affects LPS-induced cytokine production. Immature DCs were stimulated with different concentration of IMOD in the presence or absence of LPS, and cytokines were measured by real time quantitative PCR. Treatment of immature DCs with IMOD alone did not induce cytokines (data not shown). Notably, IMOD inhibited LPS-induced production of pro-inflammatory cytokines IL-6, IL-12p35, IL12p40, and TNFα at mRNA level, in a dose dependent manner (**Figures 2A,C,E,F**). Consequently the levels of LPS induced IL6 and IL12p70 were also abolished by IMOD in dose dependent manner (**Figures 2B,G**). LPS-induced TNF secretion was already strongly inhibited by the lowest IMOD concentration, which may suggest additional regulation by IMOD at the level of translation or protein stability (**Figure 2D**). In contrast the anti-inflammatory cytokine IL-10 mRNA levels induced by LPS treatment were not affected (**Figure 3A**), while there was partial reduction of LPS-induced IL10 secretion caused by the highest IMOD concentration (**Figure 3B**). These data suggest that IMOD might affect translation rate but not transcription of *il10*. Taken together those data suggest that specifically IMOD suppresses pro-inflammatory cytokines induced by LPS.

### IMOD Attenuates DC-Induced T Cell Activation

**FIGURE 4:**
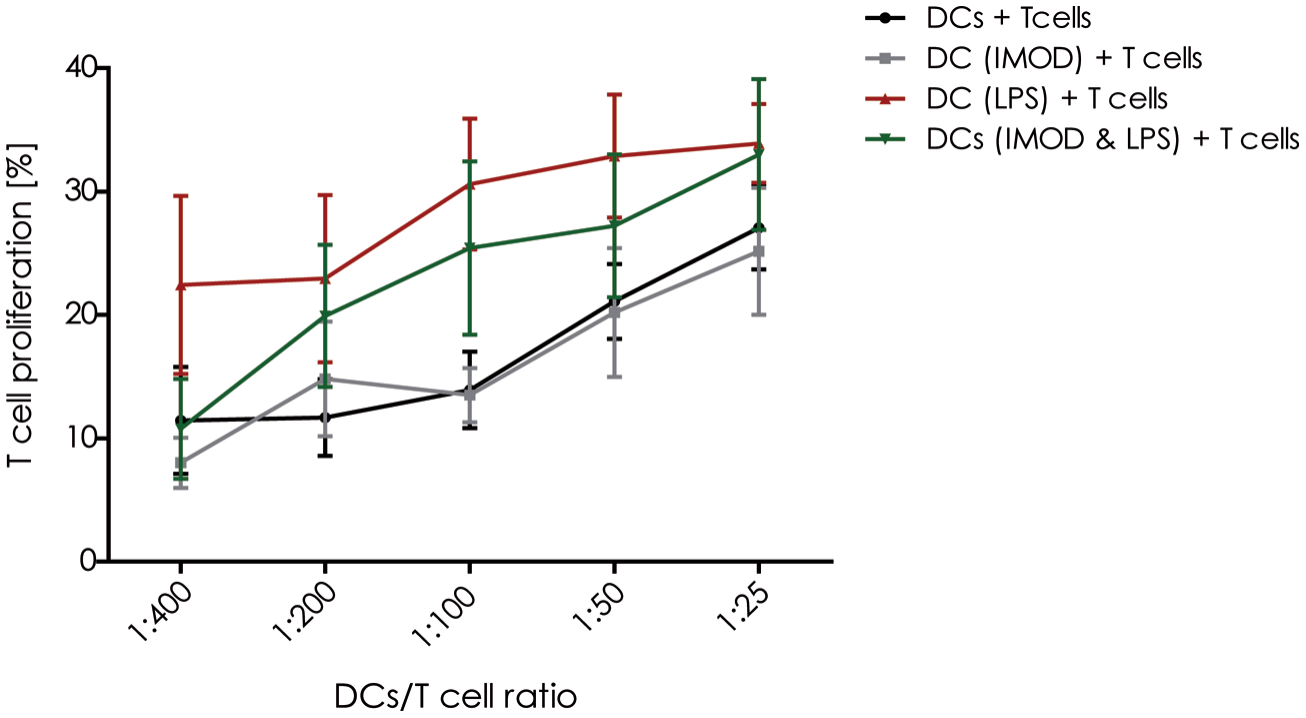
**IMOD inhibits DC-induced T cell proliferation.** Immature dendritic cells were stimulated with IMOD (concentration 1:800) in the presence and absence of LPS. After 18 h of incubation DCs were washed and cultured with allogenic PBLs (1 × 10^5^) at different ratios for 5 days. The proliferation of T lymphocytes was assessed by overnight BrdU incorporation. The results are representative of independent experiments obtained from three donors.

Activation of T lymphocyte by mature DCs is crucial to initiate an effective adaptive immune response against invading pathogens. Therefore, we investigated whether IMOD interfered with the T cell activation capacity of DCs. Our results show that IMOD treatment had a negative influence on T cell proliferation induced by LPS-stimulated DCs (**Figure 4**). Although the differences did not reach statistical significance, there was a clear trend toward lower T cell proliferation rate when stimulated with DCs activated in the presence of IMOD. IMOD alone did not influence T cell proliferation induced by immature DCs (**Figure 4**). Additionally, IMOD alone did not induce T cell proliferation or had an effect on PHA/IL2 induced proliferation (Figure S3). These data may suggest that IMOD has a potential to suppresses T cell activation capacity of LPS-activated DC.

### IMOD Treatment Skews T Helper Cell Polarization Toward Promotes T_H_2

**FIGURE 5:**
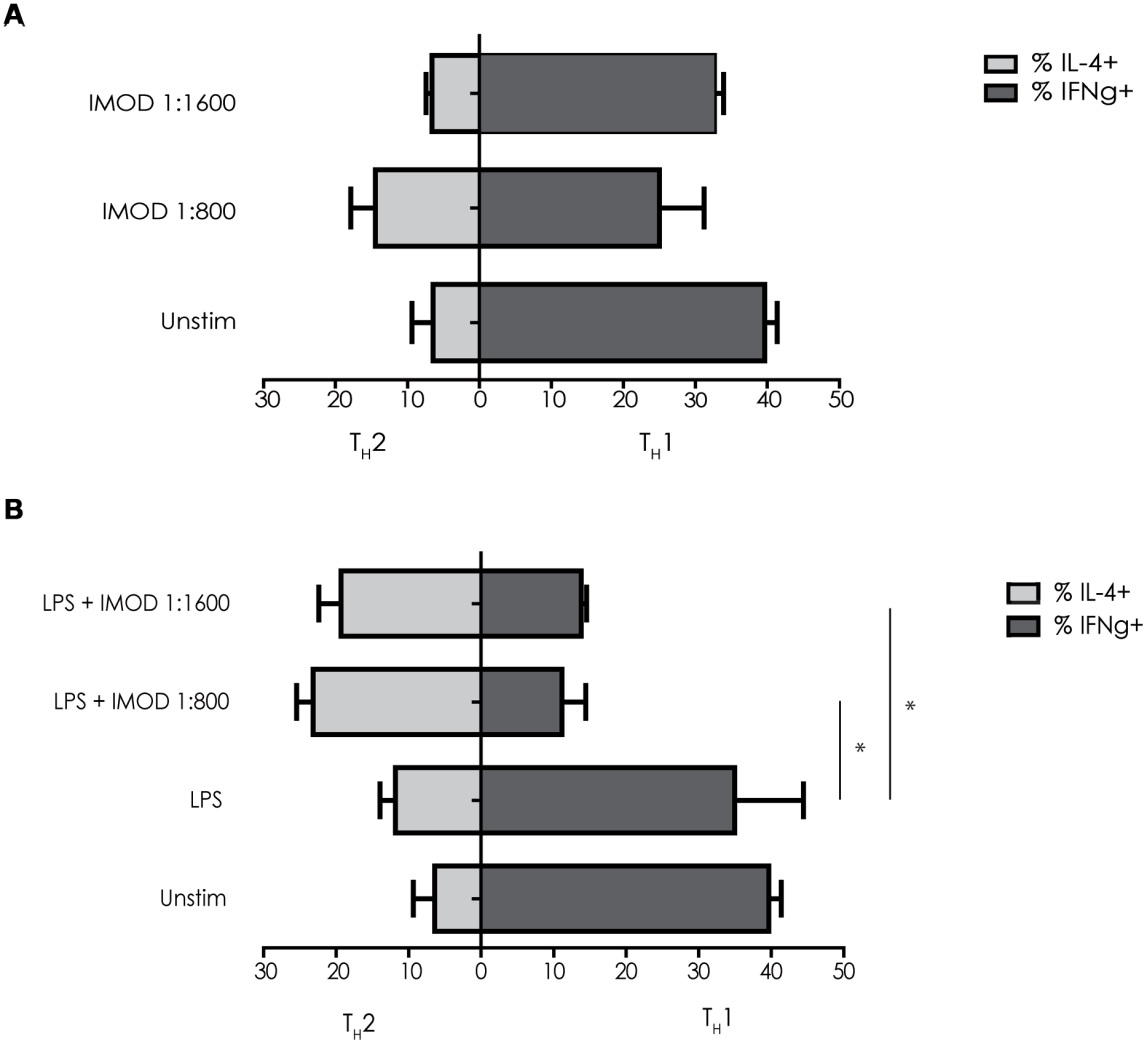
**IMOD skews T helper cell polarization toward T_**H**_2.** Immature dendritic cells were incubated for 48 h with IMOD in the presence **(B)** or absence **(A)** of LPS (10 ng/mL). Next naïve CD4^+^ T cells were added and after 13 days of co-culture the differentiation was assessed by staining for intracellular INF-γ (T_H_1) and IL4 (T_H_2) by flow cytometry. The results represented as mean ± standard deviation of three independent experiments. ^∗^*P* < 0.05; ^∗∗^*P* < 0.01. IMOD/LPS-treated vs. LPS stimulated samples.

IL-12p70 expression is necessary for T_H_1 differentiation, as inhibition of IL-12p70 skews T cells responses toward T_H_2 cytokines profile. Because IMOD inhibited production of pro-inflammatory cytokines including IL-12p70, we investigated whether IMOD modulated T helper cell polarization. DCs were stimulated with different concentrations of IMOD in presence or absence of LPS. Subsequently, DCs were washed and mixed with naïve CD4^+^ T cells and T cell polarization was investigated. Our results show that IMOD-stimulated DCs skewed T cells responses toward the T_H_2 cytokines profile (**Figure 5A**). Stimulation of DCs with LPS induced a mixed T_H_1/T_H_2 response, whereas co-treatment with IMOD suppressed induction of T_H_1 cells leading to more T_H_2 skewed response (**Figure 5B**). These data strongly suggest that IMOD skews T helper cell polarization toward T_H_2.

## Discussion

Herbal extracts have been shown to modulate immune responses during inflammation (Balzarini et al., 1992; Huang et al., 2008; Alvarez et al., 2011). Setarud (IMOD) is a natural medicine that consists of a mixture of herbal extracts including *U. dioica* (nettle), *T. vulgare* (Tansy), and *R. canina* with addition of selenium, flavonoids, and carotenes (Mohammadirad et al., 2011). The flavonoid compounds extracted from the *T. vulgare* leaf showed immunomodulatory activity (Xie et al., 2007). The ethyl acetate extract of aerial parts of *T. vulgare* and the isolated compound parthenolide possess strong anti-HSV1 activity (Alvarez et al., 2011). Furthermore, *R. canina* exhibits anti-oxidative and anti-inflammatory properties (Lattanzio et al., 2011) and its extract has been employed successfully in the number of studies in patients suffering from rheumatoid arthritis, osteoarthritis, and low back pain (Chrubasik et al., 2008). Until now IMOD has been shown to have beneficial effects in patients with severe sepsis, by lowering the levels of TNFα when compared with patients receiving only standard treatment (Mahmoodpoor et al., 2010). Furthermore, IMOD has been shown to increase CD4^+^ T cell counts in HIV infected patients (Mohraz et al., 2009, 2013). Thus, IMOD has been shown to modulate immune responses during different inflammatory disorders (Farhoudi et al., 2013) but little is known about the molecular mechanism of IMOD. Our data suggest that IMOD modulates DC function.

IMOD alone did not induce DC maturation but it strongly inhibited LPS-induced pro-inflammatory cytokines IL-6, TNFα, and IL-12p70. The inhibition of pro-inflammatory cytokines might prevent over activation of the immune system, which is further supported by the attenuation of T cell activation by IMOD-treated DC. Thus, IMOD strongly counteracts pro-inflammatory responses which might prevent immune-mediated tissue damage.

IMOD skewed T_H_ cell differentiation toward a T_H_2 response. Although induction of T_H_2 responses is affected by different cytokines and cell-surface molecules (Pulendran et al., 2010), our data suggest that IMOD-mediated suppression of IL-12 might underlie the observed T_H_2 polarization.

Although the mechanism of action is unclear, our data strongly suggest that IMOD affects TLR4 signaling and thereby prevent pro-inflammatory cytokine induction. Interestingly, components of IMOD have been shown to interact with the C-type lectin DC-SIGN and might thereby affect TLR signaling as shown for mannose- and fucose-containing carbohydrate structures (Gringhuis et al., 2014). Recently we have shown that fucose binding to DC-SIGN leads to the activation of Bcl3, which represses proinflammatory cytokine expression, while inducing IL-10 and T_H_2-attracting chemokine expression, leading to T_H_2 polarization (Gringhuis et al., 2014). Further research is required to elucidate the signaling pathway induced by IMOD that affects TLR4 signaling. TLR-mediated immune responses play an important role in a variety of diseases including infectious diseases, autoimmune diseases, and atherosclerosis (Cook et al., 2004). Therefore manipulation of TLR-triggered signaling is of wide clinical interest and the IMOD-induced signaling pathway might prove to be important in the development of novel immunotherapies.

## Conflict of Interest Statement

The authors declare that the research was conducted in the absence of any commercial or financial relationships that could be construed as a potential conflict of interest.
