# Supplementary material for: Herbal medicine IMOD suppresses LPS-induced production of proinflammatory cytokines in human dendritic cells
Source: Front Pharmacol. 2015 Mar 27;6:64. doi: 10.3389/fphar.2015.00064 (PMC4375992; doi:10.3389/fphar.2015.00064)
Supplement: Supplementary file 1 [file data_sheet_1.doc]

**Supplementary Information**

**Supplementary Materials and Methods**

**Cell viability assay**

After overnight culture of DCs in the presence of absence of different concentrations of IMOD the cell viability was assessed using CellTiter-Glo® Luminescent Cell Viability Assay (Promega, Madison, Wisconsin, USA), according to the manufacturer protocol.

**Supplementary Figure legends**

**Supplementary Figure 1. IMOD does not influence DCs viability.** Immature DCs were cultured overnight with different dilutions of IMOD and afterwards their viability was assessed by the CellTiter-Glo® Luminescent Cell Viability Assay. Staurosporine was used as a inducer of cell death.

**Supplementary Figure 2. IMOD treatment does not affect DC maturation.** Immature DCs were stimulated with different dilutions of IMOD in the presence or absence of LPS. 18 hours after stimulation, expression of maturation markers CD80 ,CD86, CD83, CD40 and HLA-DR was measured by flow cytometry. Data are representative of at least two (c,d,e) or 4 (a,b) representative experiments.

**Supplementary Figure 3. IMOD does not directly influence T cell proliferation.** Allogenic PBLs (1x 10^5^) were incubated with or without IL2/PHA in the presence or absence of IMOD (1:800) for 5 days. The proliferation of T lymphocytes was assessed by overnight BrdU incorporation. The results are representative of independent experiments obtained from 3 donors.

**Supplementary** Table 1

| **Expression primer sequences** | | |
| --- | --- | --- |
| **Gene product** | **Forward primer (5’-3’)** | **Reverse primer (5’-3’)** |
| **IL6** | TGCAATAACCACCCCTGACC | TGCGCAGAATGAGATGAGTTG |
| **TNFα** |  |  |
| **IL12p35** | CTCCAGAAGGCCAGACAAAC | AATGGTAAACAGGCCTCCACT |
| **IL12p40** | CCAGAGCAGTGAGGTCTTAGGC | TGTGAAGCAGCAAGGAGCG |
| **IL10** |  |  |
| **GAPDH** | CCATGTTCGTCATGGGTGTG | GGTGCTAAGCAGTTGGTGGTG |
