# Supplementary figures and images for: Herbal medicine IMOD suppresses LPS-induced production of proinflammatory cytokines in human dendritic cells
Source: Front Pharmacol. 2015 Mar 27;6:64. doi: 10.3389/fphar.2015.00064 (PMC4375992; doi:10.3389/fphar.2015.00064)

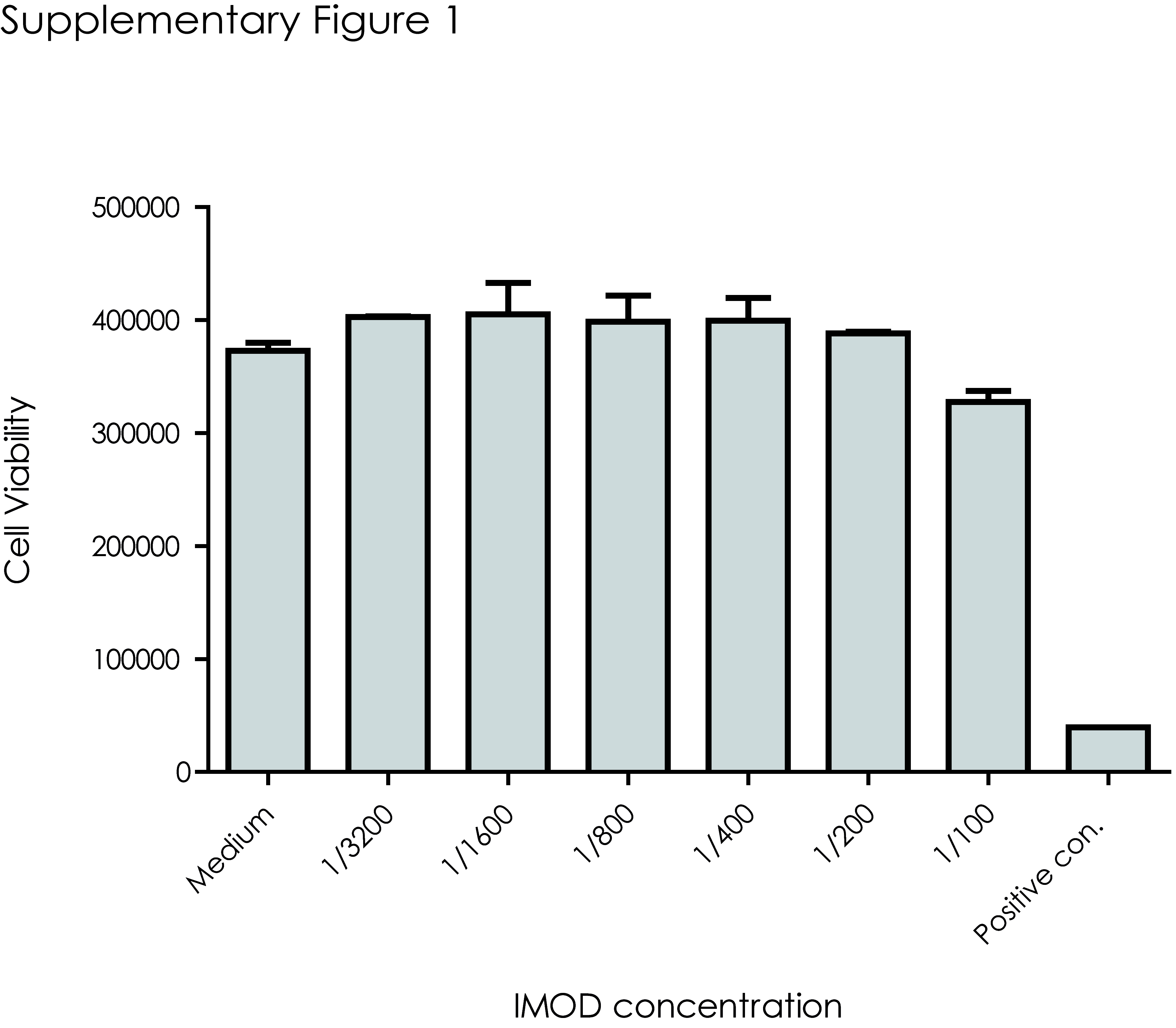

Supplement: Supplementary file 2 [file image_1.tif]

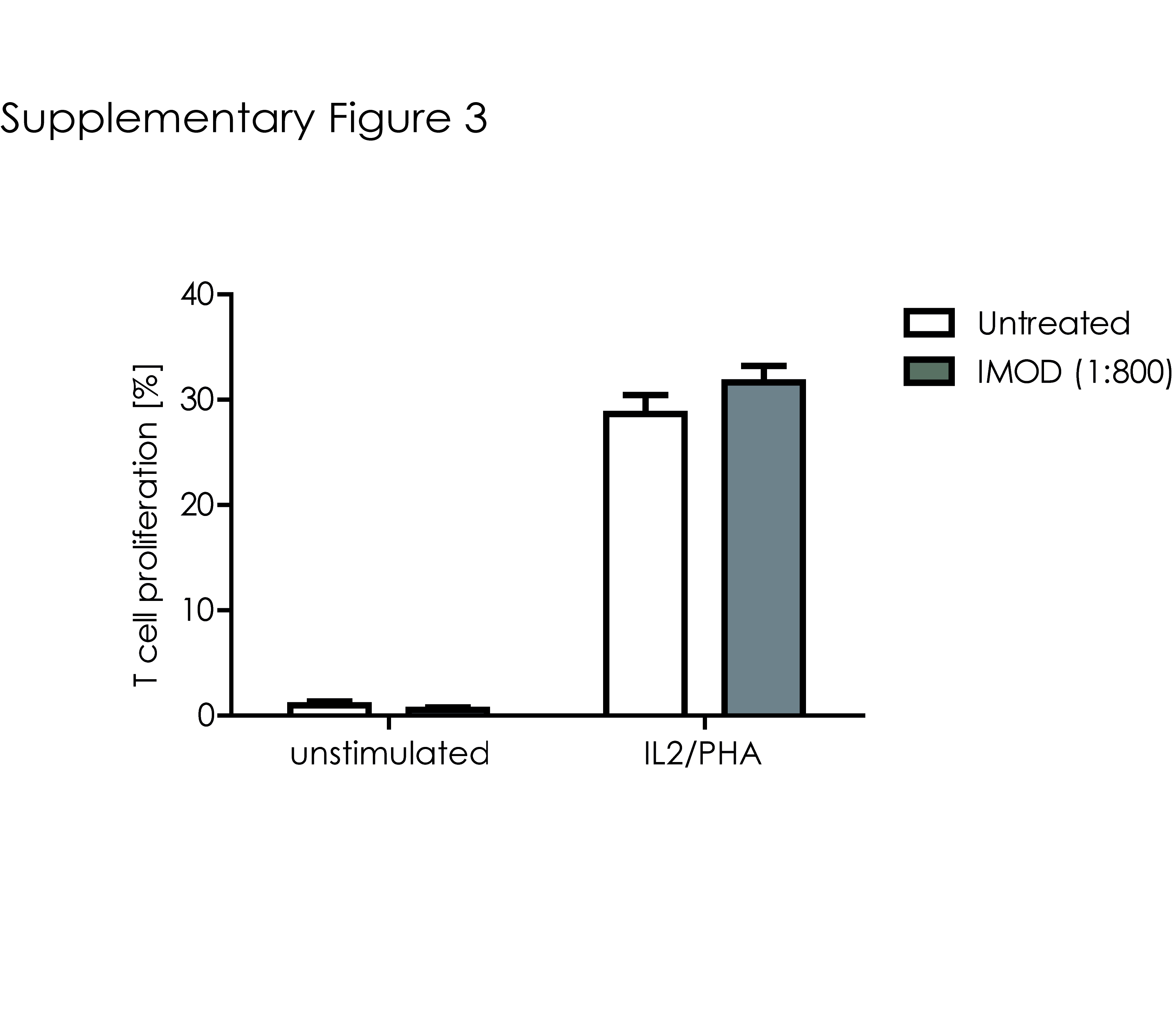

Supplement: Supplementary file 4 [file image_3.tif]
